# High Dimensional Variable Selection with Error Control

**DOI:** 10.1155/2016/8209453

**Published:** 2016-08-15

**Authors:** Sangjin Kim, Susan Halabi

**Affiliations:** Department of Biostatistics and Bioinformatics, Duke University Medical Center, Box 2717, Durham, NC 27710, USA

## Abstract

*Background.* The iterative sure independence screening (ISIS) is a popular method in selecting important variables while maintaining most of the informative variables relevant to the outcome in high throughput data. However, it not only is computationally intensive but also may cause high false discovery rate (FDR). We propose to use the FDR as a screening method to reduce the high dimension to a lower dimension as well as controlling the FDR with three popular variable selection methods: LASSO, SCAD, and MCP.* Method.* The three methods with the proposed screenings were applied to prostate cancer data with presence of metastasis as the outcome.* Results.* Simulations showed that the three variable selection methods with the proposed screenings controlled the predefined FDR and produced high area under the receiver operating characteristic curve (AUROC) scores. In applying these methods to the prostate cancer example, LASSO and MCP selected 12 and 8 genes and produced AUROC scores of 0.746 and 0.764, respectively.* Conclusions*. We demonstrated that the variable selection methods with the sequential use of FDR and ISIS not only controlled the predefined FDR in the final models but also had relatively high AUROC scores.

## 1. Introduction

Prognosis will continue to play a critical role in patient management and decision making in 21st century medicine. Advanced technologies for genomic profiling are now available and they include millions of sets of molecular data in these assays. A critical element of personalized medicine is utilizing and implementing validated diagnostic signatures (or classifiers) for diagnosing or treating cancer patients. These signatures are built and validated utilizing common statistical methods and machine learning tools. For example, the Decipher signature has been developed as a prognostic model to predict metastasis after radical prostatectomy in patients with prostate cancer [1]. The Decipher score is a 22-feature genomic classifier that has been used to predict metastasis and has been independently validated for prediction of prostate metastasis [2–5]. Another example is oncotypeDx that has been used to stratify randomization and guide treatment in women with breast cancer [6].

A vital step in model building is data reduction. It is assumed that there are several variables that are associated with the clinical outcome in the large dimensional data. The main purpose of the variable selection is to detect only those variables related to the response. Variable selection is composed of two steps: screening and model building. The screening step is to reduce the large number of variables into moderate size while maintaining most of the informative variables relevant to the clinical response. In contrast, in the model building step, investigators develop a single best model utilizing a proper evaluation criterion.

Penalized variable selection methods have played a key role in identifying important prognostic models in several areas in oncology [7–9]. Many articles focused on the development of methodologies related to “small N and large P” with the advent of high throughput technology in cancer. The sure independence screening (SIS) was introduced to reduce the high dimension to below the sample size to efficiently select the best subset of variables to predict clinical responses [10]. Although this approach is popular, it does not perform well under some situations. First, unimportant variables that are heavily correlated with important variables are more highly likely to be selected than important variables that are weakly associated with the response. Second, important variables that are not marginally significantly related to the response are screened out. Finally, there may be collinearity between variables that may impact the calculations of the individual predictors.

The iterative sure independence screening (ISIS) was proposed to overcome the above issues. The procedure is to apply iteratively high dimensional variable screening followed by the proper scale of variable selection until the best subset of variables with high predictive accuracy is obtained. ISIS screening, however, is also computationally intensive and leads to high false discovery rate (FDR) in ultra-high dimensional setting (*P* ≫ 1 mils).

The oncology literature is rich in articles related to the use of validated signatures. Despite their abundance, comparisons and the performance of these various methods have not been studied. We propose to use the false discovery rate (FDR) of the multiple testing correction methods as a screening method to reduce the high dimension to lower dimension as well as controlling the false discovery rate in the final model. We investigate the feasibility of the sequential use of FDR screening method with the ISIS and utilize three popular variable selection methods: LASSO [11], SCAD [12, 13], and MCP [14], through the extensive simulation studies. To the best of our knowledge, this is the first paper that thoroughly analyzes and compares the performance of the variable selection methods with the sequential use of FDR and ISIS screening methods. We use a prostate cancer signature as an example [1] where the number of probes is around 1.4 million and the clinical outcome is binary in nature: presence of metastasis (presence of metastasis = 1, no metastasis = 0) by fitting models based on the simulation results.

In addition, we provide a broad review of the existing penalized variable selection methods with screening methods. The remainder of this paper is organized as follows. In Section 2, we provide general details of the screening methods of FDR [15] and ISIS [10] and the variable selection methods with the penalized logistic regression. In Section 3, we describe the simulation studies and in Section 4, we summarize the results of the simulations. We then apply the best screening methods from the simulation studies to the real data in Section 5. Finally in Section 6, we discuss our findings.

## 2. Methods

We divide this section into several subsections describing the methods used in our paper. The screening section briefly discusses commonly used methods that reduce high dimensionality: false discovery rate (FDR) and iterative sure independence screening (ISIS). We then describe the methods needed to assess variable selection models. The final section considers three existing popular variable selection methods with the logistic regression. All simulations and calculations were carried out using glmnet and ISIS packages in the R library, and the code is available at https://www.duke.edu/halab001/FDR.

### 2.1. Benjamini and Hochberg False Discovery Rate (FDR)

The false discovery rate is defined as the expected proportion of incorrectly rejected null hypotheses. That is,

(1) $$\begin{array}{c} E\left(\;\frac{V}{R}\;|\;R>0\;\right), \end{array}$$

where *V* is the number of falsely rejected hypotheses and *R* is the total number of rejected hypotheses. We focus on the Benjamini and Hochberg FDR [15] method as a screening method in the simulation studies and application. Briefly, the procedure works as follows. Let *q* denote the FDR, where *q* ∈ (0,1).

(1) Let *p*_1_,…, *p*_*m*_ be the *p* values of the *m* hypothesis tests and sort them from smallest to largest. Denote these ordered *p* values by *p*_(1)_,…, *p*_(*m*)_.
(2) Let $\hat{k}=max\left\{k:p_{\left(k\right)}\leq \left(k\times q\right)/m\right\}$,  *k* = 1,2,…, *m*.
 If $\hat{k}>1$, then reject *p*_(1)_,…, *p*_(*k*)_ and if $\hat{k}=0$, then there is no rejection of the *m* hypothesis.

### 2.2. Iterative Sure Independence Screening (ISIS)

The ISIS method was proposed to overcome the difficulties caused by the sure independence screening [16]. Briefly, the algorithm works in the following way:

(1) The likelihood of marginal logistic regression (LMLR) is computed for every *j* ∈ *S* = {1,2,…, *p*}. Then *d* which is *N*/4log⁡(*N*) of the top ranked variables of the descending order list of the LMLR is selected to obtain the index set ${\hat{I}}_{1}$.
(2) Apply those variables in ${\hat{I}}_{1}$ to the penalized logistic models to obtain a subset of indices ${\hat{M}}_{1}$.
(3) For every variable $j\in \{S-{\hat{M}}_{1}\}$, the likelihood of the marginal logistic regression condition on the variables in ${\hat{M}}_{1}$ is solved. Then the likelihood estimators are sorted in descending order and then the *d* top ranked variables are selected to get the index set ${\hat{I}}_{2}$.
(4) Apply those variables in ${\hat{I}}_{2}\cup {\hat{M}}_{1}$ to the penalized logistic models to obtain a new index set ${\hat{M}}_{2}$.
(5) Steps (3) and (4) are repeated until ${\hat{M}}_{l}$ = *d* or ${\hat{M}}_{l}={\hat{M}}_{l-1}$.

### 2.3. Regularizing Methods with Penalized Logistic Regression

The logistic regression is one of the most commonly used methods for assessing the relationship between a binary outcome and a set of covariates and building prognostic models of clinical outcomes. In addition, it is widely used in the classification of two classes such as the development of metastasis in prostate cancer [1]. The purpose of variable selection with the logistic regression model in high dimensional setting is to select the optimal subset of variables that will improve the prediction accuracy [17]. Variable selection in high dimensional setting is composed of two components: a likelihood function and a penalty function in order to obtain better estimates for prediction.

Let the covariates of *i*th individual be denoted as *x*_*i*_ = (*x*_*i*1_,…,*x*_*ip*_)^*T*^  for  *i* = 1,…, *N* and *p* is the total number of covariates. The penalized logistic regression is as follows:

(2) −1N∑i=1Nyilog⁡pi+1−yilog⁡1−pi+pβ,i=1,2,…,N,

where *p*(*β*), a penalty, is function and *y*_*i*_ is 1 for cases and 0 for controls. The probability that *i*th individual is a case based on covariates' information is expressed as

(3) $$\begin{array}{c} p_{i}=\frac{\exp\left(x_{i}^{\prime }\beta \right)}{1+\exp\left(x_{i}^{\prime }\beta \right)},\;i=1,2,\ldots ,N. \end{array}$$

The regression coefficients are obtained by minimizing the objective function (2).

One of the most popular penalty functions is the least absolute shrinkage and selection operator (LASSO) [11]. It forces the coefficients of unimportant variables to be set to 0 and then the LASSO has sparsity property. The LASSO estimates are obtained by minimizing the above penalized logistic regression form (2). It has a satisfactory performance in identifying a small number of representative variables. Though LASSO is widely used in most applications [18–21], its robustness is open to question as it has the tendency to randomly select one of the variables with high correlation and exclude the rest of the predictors [22]. Another disadvantage of LASSO is that it always chooses at most *N* (sample size) number of predictors even though there are more than *N* variables with true nonzero coefficients [23]. The coefficients estimates are obtained by minimizing the following objective function based on the likelihood function of logistic regression:

(4) β^lasso=argminβ⁡−1N·∑i=1Nyilog⁡pi+1−yilog⁡1−pi+λ∑j=1pβj.

Another method commonly employed is the smoothly clipped absolute deviation (SCAD) with a concave penalty function that overcomes some of the limitations of the LASSO [12]. The coefficients from SCAD are solved by minimizing the following objective function:

(5) β^scad=argminβ⁡−1N·∑i=1Nyilog⁡pi+1−yilog⁡1−pi+∑i=1pfλ,γβj.

The SCAD penalty function, *f*_*λ*,*γ*_(*β*_*j*_), is defined by

(6) fλ,γβj=λγβjI0≤βj≤λ+λγβj−λ−βj2−λ2/2a−1+λ2·Iλ<βj≤λγ+γ+1λ22

with *λ* ≥ 0 and *γ* > 2.

The minimum concave penalty (MCP) is also a recognized method with SCAD, where the coefficients are estimated via minimization of the following objective function:

(7) β^mcp=argminβ⁡−1N·∑i=1Nyilog⁡pi+1−yilog⁡1−pi+∑i=1pfλ,γβj.

The MCP penalty function, *f*_*λ*,*γ*_(*β*), is defined by

(8) fλ,γβ=2λγβj−βj22γIβj≤λγ+λ2γ2Iβj>λγ,

for *λ* ≥ 0 and *γ* > 1.

## 3. Simulation Studies

### 3.1. Simulation Setup

**Figure 1:**
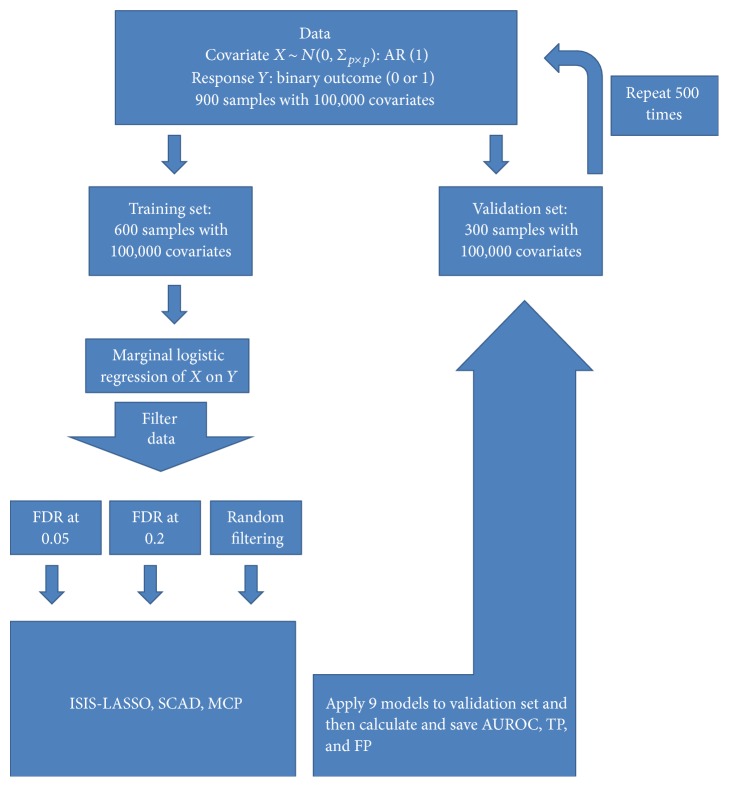
Diagram showing simulation procedures.

We performed extensive simulation studies to explore the performance of three popular variable selection methods: LASSO, SCAD, and MCP in high dimensional setting. We employed 10-fold cross validation to tune the regularization parameter for the methods.  Figure 1 describes the schema of the simulation procedures.

Based on the logistic regression model, we generated the binary outcome and covariates for each simulation as follows. First, we generated *z*_1_, *z*_2_,…, *z*_*p*_ independently from *N*(0,1), and each of *z*_*i*_ is an *N* × 1 vector. We defined *x*_1_ = *z*_1_, $x_{i}=\rho x_{i-1}+\sqrt{\left(1-\rho^{2}\right)}z_{i}$, where *i* = 2,…, *p*, so that correlation of *x*_*k*_ and *x*_*l*_ was *ρ* ^|*k*−*l*|^ for some *ρ* ∈ [0,1). That is, the covariates were generated with serialized correlation structure (AR (1)). Next, we specified the true regression coefficients *β*. We fixed all of *β*'s except the first 25 *β*'s to be 0. The true nonzero *β*'s were sampled independently from uniform distribution [−1.5,2]. We considered 25 true effects of the regression coefficients since several classifiers including the Decipher score had selected 20–25 genes [1, 2] and because that number predicted reasonably well the outcome. The number of variables was fixed at *P* = 100,000, and the sample size was set at *N* = 900. Finally, the corresponding binary response *y*_*i*_ was simulated based on the Bernoulli distribution with the following:

(9) $$\begin{array}{c} y_{i}\sim Bern\left(\;p\left(\;x_{i}\;\right)\;\right),\;p\left(x_{i}\right)=\frac{e^{x_{i}\beta }}{1+e^{x_{i}\beta }}, \end{array}$$

where *x*_*i*_ = (*x*_*i*,1_, *x*_*i*,2_,…, *x*_*i*,*p*_) and *β* = (*β*_1_, *β*_2_, …,*β*_*p*_)′. Covariates were generated until the target number of 450 cases and 450 controls was reached.

We considered different simulation scenarios for the correlation matrix Σ_*P*×*P*_, *ρ* = {0, 0.1, 0.4} among variables. Each simulation scenario was composed of the nine different models with the combination of the FDR, the ISIS, and the random filtering (RF_1000_). The RF_1000_ selected 1,000 variables with the smallest unadjusted *p* values obtained from the marginal logistic regression with the three variable selection methods (LASSO, SCAD, and MCP). The reason we used RF_1000_ from the 100,000 potential variables was that the number of false discovery rates is low relative to the other random filtering (such as 2,000 or higher). Therefore, we considered the top 1,000 variables to be a reasonable number of variables screened as reference to be compared with our proposed methods.

We then simulated the data 500 times because of computational intensity. In each simulation, we randomly divided the data into two parts: the training set (*N* = 600) for model selection and the testing set (*N* = 300) for validation.

### 3.2. Metrics of Performance

We calculated the true positive rate (TP), the false positive rate (FP), the false discovery rate (FDR), the average number of false positives in the final model, the average model size, the average of the area under receiver operating characteristic (AUROC), and the number of screened true important variables through the FDR and the RF_1000_ to assess the impact of the FDR-ISIS screening method with the three variable selection methods.

The true positive rate (TP), also called sensitivity, is the proportion of positives that are identified correctly given true positives:

(10) $$\begin{array}{c} True\;\;Positive\;\;rate\;\;\left(\;TP\;\right)=\frac{TP}{\left(TP+FN\right)}, \end{array}$$

where TP is the number of the true positives and FN is the number of false negatives. The false positive rate is the proportion of incorrect identification as a true positive given true negatives. That is,

(11) $$\begin{array}{c} False\;\;Positive\;\;rate\;\;\left(\;FP\;\right)=\frac{FP}{\left(TN+FP\right)}, \end{array}$$

where the FP is the number of false positives and the TN is the number of true negatives. In addition, the average number of false positives (ANFP) was computed as the number of false positives that were selected in the final model out of 500 simulations. Furthermore, the average model size was computed as the number of variables selected in the final model out of 500 simulations.

Finally, the AUROC was utilized as a measure of the performance of the logistic regression and is the proportion of the time which a model predicts correctly given observations of a random positive and negative. A perfect model produces an AUROC = 1 whereas a random model has an AUROC = 0.5.

## 4. Simulation Results 

We summarized the simulation results for *ρ* = 0.1, one of three correlation structures in Table 1, where all 25 important covariates were assumed to have linear effects. Table 1 presents the performance of the nine different models with the FDR, ISIS, and random filtering based on 500 simulations. The average true positive rates (TP) were 0.223 and 0.268 for the three variable selection methods using FDR_.05_ − ISIS and FDR_.20_ − ISIS. The average true positive rates (TP) of the LASSO, SCAD, and MCP with RF_1000_ − ISIS were 0.46013, 0.46365, and 0.46739, respectively. These values were much higher than the two FDR screening methods which were below 0.30. On the other hand, the three variable selection methods with RF_1000_ − ISIS selected several of the false positive variables that consequently increased the false positive rate (FP). LASSO, SCAD, and MCP with RF_1000_ − ISIS included a higher average number of the false positives of 12.364, 12.260, and 12.170, respectively. Although the FDR filtering method did not select a higher number of true important variables, this screening method reduced the false positive rates below the predefined target *α*.

The average numbers of the false positives in the final models with the FDR − ISIS methods were much smaller than that of using RF_1000_ − ISIS (Table 1). Specifically, the average numbers of the false positives in the final models with the LASSO, SCAD, and MCP with FDR_.05_ − ISIS were 0.216, 0.214, and 0.214 with the corresponding standard deviations 0.0219, 0.0215, and 0.0215. As expected, the three variable selection methods with RF_1000_ − ISIS had selected a higher average model size of 22.8 than the FDR methods. Similar results were observed for FDR_.20_ − ISIS. We also calculated the false discovery rate. The variable selection models with the FDR at the target *α* = 0.05 and *α* = 0.20 controlled the false discovery rate below *α* whereas over 40% of the finally selected variables were incorrectly selected using the random filtering methods. The average AUROC scores with RF_1000_ − ISIS were relatively higher than the FDR_.05_ − ISIS and FDR_.20_ − ISIS. Similar results were noted for independent and moderate correlation *ρ* = {0,0.4} as presented in Tables S1 and S2 in Supplementary Material available online at http://dx.doi.org/10.1155/2016/8209453.

**Figure 2:**
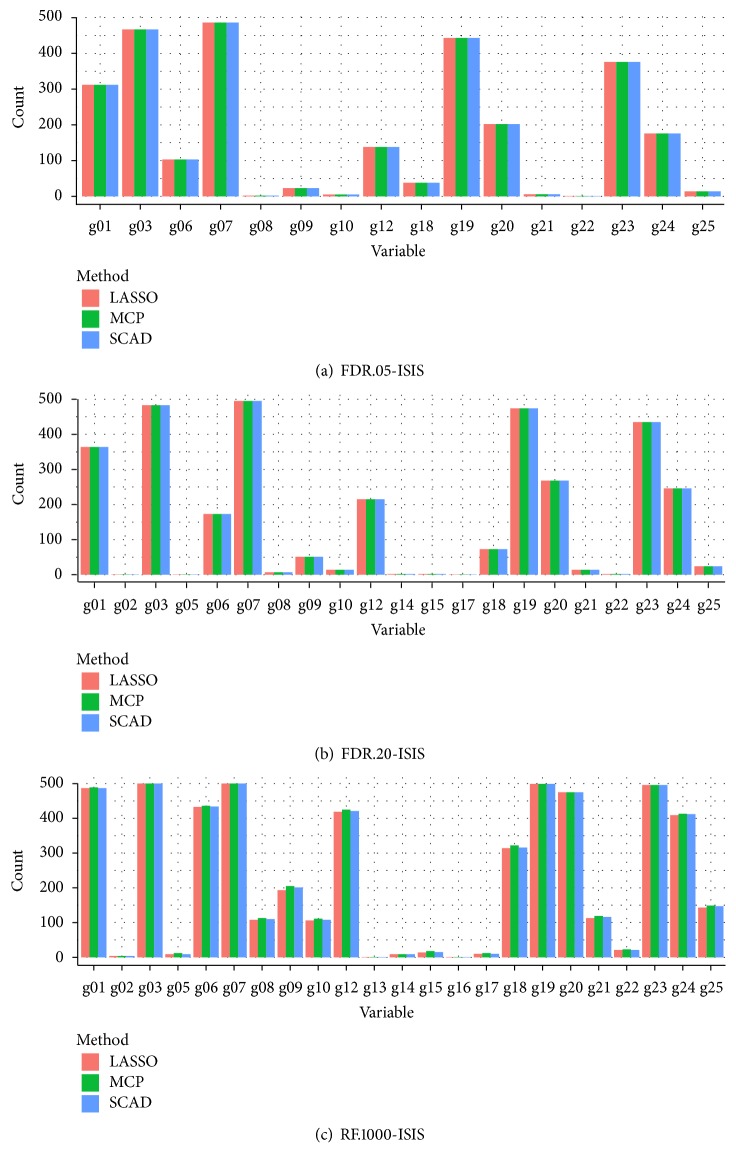
Selection frequencies of each of the 25 variables across the LASSO, the SCAD, and the MCP during 500 simulations with *ρ* = 0.1. The *x*-axis depicts the names of the variables, and the *y*-axis is the frequency of variables selected out of 500 simulations. The variables not depicted on the *x*-axis in Figure 2 did not have any counts. Each of the three methods is identified by color in legend.

Figure 2 presents the selection frequency for the 25 important variables under the three different screening methods. The *x*-axis denotes the variable name and the *y*-axis represents the frequency of selection out of 500 simulations. The variables not depicted on the *x*-axis in Figure 2 did not have any counts and thus were not selected in the simulation. The variables with the highest selection frequencies had true regression coefficients that were strongly associated with the clinical response. These variables were g07, g03, g19, g23, g01, g24, g12, and g06 and were selected over 100 times out of 500 simulations with average corresponding regression coefficients of 1.65, −1.45, 1.57, −1.17, 1.12, 0.968, 0.963, and −1.01 (see Table S3 in Supplementary File). The coefficients of the eight variables were ranked the highest among the 25 absolute values of the true regression coefficients which had strong effects on the response. There were no differences in selecting the important variables by the variable selection methods (LASSO, SCAD, and MCP). In addition, similar patterns of the selection frequencies were observed for both FDR_.05_ − ISIS and FDR_.20_ − ISIS as shown in Figures 2(a) and 2(b) while Figure 2(c) showed a little variation with RF_1000_ − ISIS. The results were similar for independent and moderate correlation *ρ* = {0,0.4} (Figures S1 and S2 in Supplementary File).

**Figure 3:**
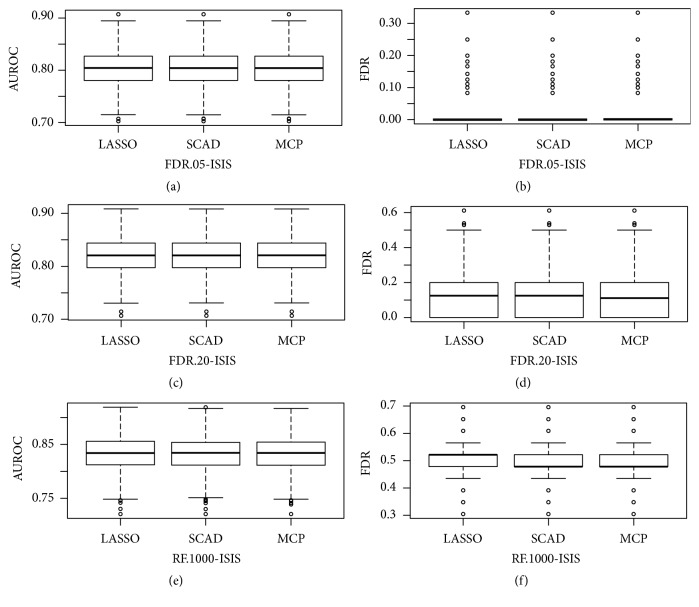
(a, c, e) The AUROC scores under *ρ* = 0.1. The *x*-axis is the name of methods and *y*-axis is AUROC scores. (b, d, f) The corresponding mean proportion of falsely selected variables in the model. The *x*-axis is the name of methods and the *y*-axis is the false discovery rate.

To gain more insights into the comparisons of the methods, we present the plots of the AUROC scores and the corresponding false discovery rate under *ρ* = 0.1 in Figure 3. (a), (c), and (e) in Figure 3 represent the AUROC scores whereas (b), (d), and (f) represent the false discovery rates using three different screening methods. The variable selection methods with random filtering screening had relatively higher AUROC scores compared to the FDR methods. However, there were a number of false positive in the final models as seen in Figure 3(f). It is noteworthy that the variable selection methods using the FDR not only controlled the FDR below the target *α* = 0.05 and *α* = 0.20 but also had AUROC scores that were relatively high (Figures 3(d) and 3(e)). Similar patterns were observed for independent and moderate correlation *ρ* = {0,0.4} (Figures S3 and S4 in Supplementary File).

Therefore, the FDR − ISIS screening method is preferred to RF_1000_ − ISIS since it allowed the variable selection methods to obtain the proper AUROC scores while controlling the false discovery rate at the nominal level of *α*. As a result of the simulation studies, we applied the three variable selection methods with the FDR and ISIS screening to the high dimensional data of the prostate cancer in the following section.

## 5. Real Data Analysis 

**Figure 4:**
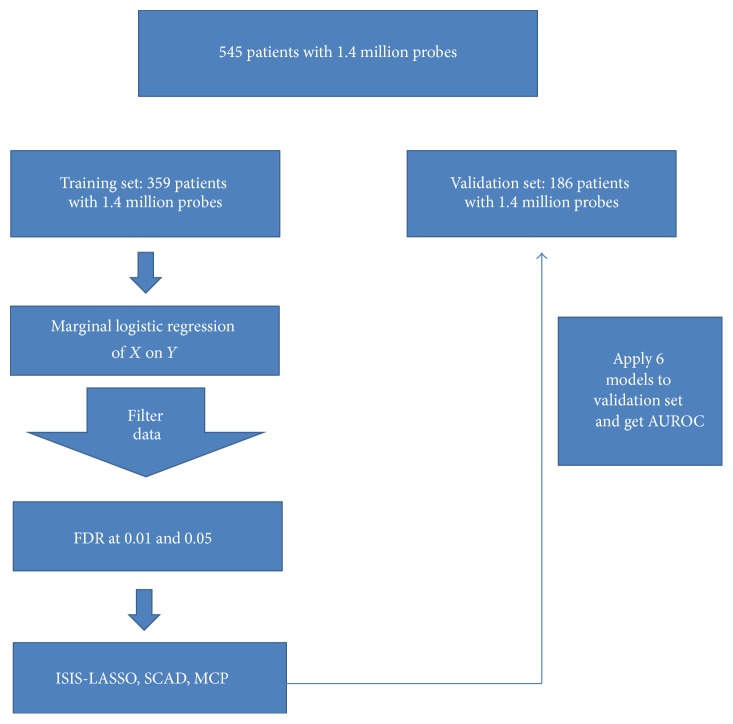
The schema of prognostic model building for the prostate cancer.

We analyzed the prostate cancer data from the public domain (http://www.ncbi.nlm.nih.gov/geo/query/acc.cgi?acc=GSE46691: GSE46691). The dataset has 1.4 million probes and the primary outcome is presence of metastasis (yes or no) by fitting the LASSO, SCAD, and MCP methods using the FDR and ISIS screenings suggested from the simulation studies with the sequential filtering of both FDR and ISIS. In the prostate cancer application, we considered the false discovery rate (FDR) at *α* = 0.01 as the screening method.  Figure 4 describes the schema of the prognostic model building for the prostate cancer. We utilized the training set that was obtained from the random split and was composed of 359 individuals (140 cases and 219 controls) with 1.4 million probes to build each of the three models. We then estimated the AUROC scores with the validation set with 186 individuals (72 cases and 114 controls). We used 10-fold cross validation for each of the variable selection models to tune the parameters after the screening. We obtained 39 variables with FDR at *α* = 0.01. We repeated each of the three models 100 times to improve the AUROC with those screened variables.

**Figure 5:**
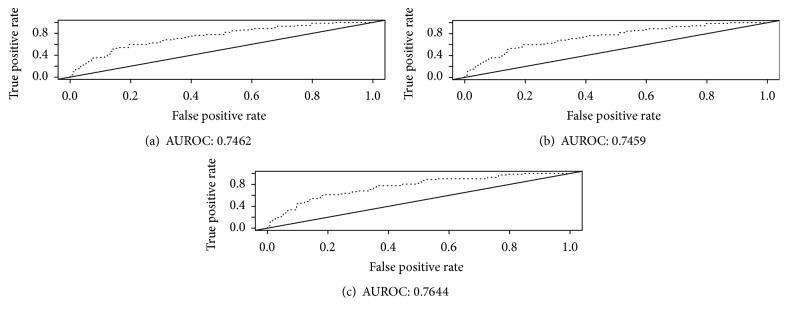
AUROC plots of using the LASSO, SCAD, and MCP with the screenings of both FDR at 0.01 and ISIS. (a), (b), and (c) are for the LASSO, the SCAD, and the MCP variable selection methods, respectively.

Figure 5 shows the AUROC plots of the three models. Based on FDR at *α* = 0.01, the LASSO, SCAD, and MCP identified 12 genes (CAMK2N1, AN07, RPL7A, MALAT1, MYBPC1, TMP0, UBE2C, DID01, RAB25, LOC728875, FTH1, and MKI67), 11 genes (CAMK2N1, AN07, RPL7A, MALAT1, MYBPC1, TMP0, UBE2C, DID01, RAB25, LOC728875, and FTH1), and 8 genes (CAMK2N1, AN07, RPL7A, MALAT1, MYBPC1, TMP0, UBE2C, and DID01) gene models out of 39 potential variables with AUROC scores of 0.746 (95% CI = 0.675–0.818), 0.746 (95% CI = 0.674–0.817), and 0.764 (95% CI = 0.695–0.834), respectively (refer to Table S4 for more details in Supplementary File). It is noteworthy to note that MCP selected the same set of genes as SCAD and LASSO and the 95% confidence intervals were overlapping. On the other hand, using the FDR at *α* = 0.05, LASSO, SCAD, and MCP selected 15, 13, and 15 gene models out of 565 potential genes with corresponding AUROC scores of 0.697 (95% CI = 0.619–0.775), 0.714 (95% CI = 0.637–0.791), and 0.683 (95% CI = 0.603–0.763), respectively. It is worthwhile to note that MCP had the highest AUROC score (FDR-ISIS at *α* = 0.01 and AUROC = 0.764) followed by LASSO (FDR-ISIS at *α* = 0.01 and AUROC = 0.746) although the results were not consistent with the FDR at *α* = 0.05. This could be due to the larger number of potential variables (565 variables) when using FDR at a higher level. Nevertheless because our interest was to use FDR at *α* = 0.01, MCP and LASSO methods were used for the variable selection in our real example.

Table 2 presents the selected probes and their corresponding genes from the two models that had two highest AUROC scores among the six models. LASSO and MCP identified each of the 12 and 8 genes that were associated with developing prostate cancer metastasis. The four genes (ANO7, UBE2C, MYBPC1, and CAM2KN1) associated with developing prostate cancer metastasis were detected in both models. These four genes were a subset of the 22 biomarkers for the Decipher PCa classifier [1]. MYBPC1 (Myosin-Binding Protein C) on chromosome 12 and ANO7 (Anoctamin 7) on chromosome 2 were only downregulated genes whereas the other 10 genes including UBE2C (Ubiquitin-Conjugating Enzyme E2C) on chromosome 20 and CAMK2N1 (Calcium/Calmodulin-Dependent Protein Kinase II Inhibitor 1) on chromosome 1 were the top upregulated genes as presented in Figure S5.

## 6. Discussion

This paper explored the feasibility of using the false discovery rate (FDR) followed by ISIS as screening methods in conjunction with three popular variable selection methods in ultra-high dimensional data for the purpose of controlling FDR and improving AUROC scores.

Our simulation studies demonstrated that the variable selection methods with FDR − ISIS not only controlled the false discovery rate below the target *α*, but also produced high AUROC scores. Furthermore, the results showed that the false discovery rate was controlled conservatively even with the increased correlation structures. As demonstrated in the simulation studies, if the truly prominent variables have not passed through the screening, they would lose the opportunity to be selected to the final model. Thus, the prediction accuracy may be relatively reduced. Currently, most multiple testing correction methods underscore the priority of identifying prominent variables. Therefore, effective filtering techniques are ultimately needed for the situation when there are weak effects among the important variables.

Although RF_1000_ − ISIS produced the highest AUROC through the simulation studies, it also had the highest false discovery rate. It is reasonable to expect that if one variable is not selected during the screening step, then the other variables that were correlated with the unselected variable have a tendency not to be chosen in the final model. As expected, the true positive rates of RF_1000_ − ISIS were relatively higher than those of using FDR − ISIS. This is because random filtering had more opportunity to select the true important variables. Due to the relatively high number of true important variables selected, the number of unimportant variables highly correlated with the true important variables was also high. This may explain why the average AUROC scores were highest in our simulation studies for RF_1000_ − ISIS.

There are some caveats to the sequential use of FDR with ISIS. First, the total number of true important variables was restricted to 25 variables in the simulation studies. This may explain why the three variable selection methods, LASSO, SCAD, and MCP, performed similarly in the simulations. Second, the computational time for 500 simulations with ISIS alone was 52,500 minutes (around 36.45 days). Although ISIS is computationally intensive, FDR with ISIS performed very well and it took 9,625 minutes to perform the 500 simulations (7 days).

Turning back to our motivation example of prostate cancer, LASSO and MCP under the FDR_0.01_ − ISIS screening methods produced the best AUROC scores. We also present the results for other LASSO and MCP methods selected 12 and 8 genes out of 39 screened probes using the FDR at the target *α* = 0.01 and had the AUROC scores of 0.7462 and 0.7644, respectively. The AUROC score of the MCP was 0.144 points higher than what was reported by Erho et al. ([1]; AUC = 0.75). Although the authors did not report a 95% confidence interval for the AUROC scores, it is most likely that the 95% confidence intervals for the AUROC scores of Erho et al. and the MCP were overlapping.

In summary, based on our extensive simulations, FDR with ISIS seems to be superior to random filtering in terms of error control and is less computationally intensive compared with ISIS only. We also showed that the classifier based on 8 genes detected by the MCP had similar performance to the prognosis for early clinical metastasis prostate cancer model. To our knowledge, this is the first paper that systematically compared the performance of high dimensional methods with screening methods. Based on the extensive simulation studies, effective screening procedures with penalized logistic regression methods would not only lead to controlling the FDR but also produce high area under receiver operating characteristic curve.

## Supplementary Material

Supplementary file is composed of the six parts of additional information that were not explained in the manuscript. Table S1 and S2 are the comparison of the performance for random filtering screening and FDR screenings with independent and moderate correlation ρ = {0, 0.4}, respectively in the simulation studies. Table S3 represents average true regression coefficients for the 25 variables through 500 replicates. Table S4 is comparison of area under the curve (AUC) and the corresponding 95% confidence interval with three popular variable selection methods (LASSO, SCAD, and MCP) for random screenings (1000,2000, and 4000) and FDR screenings at α = 0.01,0.05, and 0.20. Figure S1 and S2 shows the selection frequencies of each of the 25 variables across the LASSO, SCAD, and the MCP during 500 simulations with independent and moderate correlation ρ = {0, 0.4}. Figure S3 and S4 present AUC scores and the corresponding mean false discovery rate during the simulations with ρ = {0, 0.4}. Finally, Figure S5 shows boxplots of the expressions of 12 identified genes on metastasis in the prostate cancer.

## Figures and Tables

**Table 1:** The true positive rate (TP), the false positive rate (FP), and the false discovery rate (FDR), the average number of false positives (ANFP) in the final models, the final model size (size), the area under the curve (AUROC), and the number of filtered truly important variables from FDR and RF (# filter) under the low correlation coefficients, *ρ* = 0.1 among variables.

Screening	Method	TP	FP	FDR	ANFP	Size	AUROC	# filter
FDR_.05_-ISIS	LASSO	0.22336 (0.00203)	2.2*e* − 06 (0)	0.03086 (0.00303)	0.216 (0.02199)	5.8 (0.05816)	0.80285 (0.00157)	5.826 (0.05985)
	SCAD	0.22336 (0.00203)	2.1*e* − 06 (0)	0.03071 (0.003)	0.214 (0.02157)	5.798 (0.05791)	0.80286 (0.00157)	
	MCP	0.22336 (0.00203)	2.1*e* − 06 (0)	0.03071 (0.003)	0.214 (0.02157)	5.798 (0.05791)	0.80286 (0.00157)	

FDR_.20_-ISIS	LASSO	0.2676 (0.00229)	1.17*e* − 05 (0)	0.12618 (0.0055)	1.17 (0.05914)	7.86 (0.09344)	0.81965 (0.00154)	7.904 (0.09499)
	SCAD	0.2676 (0.00229)	1.16*e* − 05 (0)	0.12522 (0.00547)	1.158 (0.05866)	7.848 (0.09288)	0.81964 (0.00154)	
	MCP	0.2676 (0.00229)	1.13*e* − 05 (0)	0.12355 (0.00541)	1.134 (0.05698)	7.824 (0.09096)	0.81967 (0.00154)	

RF_1000_-ISIS	LASSO	0.42112 (0.00236)	0.0001237 (0)	0.53987 (0.00265)	12.364 (0.06298)	22.892 (0.01389)	0.83244 (0.00149)	13.286 (0.06807)
	SCAD	0.42336 (0.00243)	0.0001226 (0)	0.53635 (0.00276)	12.26 (0.06622)	22.844 (0.01624)	0.83196 (0.00147)	
	MCP	0.42656 (0.00249)	0.0001217 (0)	0.53261 (0.00283)	12.17 (0.06797)	22.834 (0.01666)	0.83191 (0.00147)	

( ): standard deviation.

**Table 2:** Probes and corresponding genes identified by the LASSO and MCP methods with FDR at *α* = 0.01 for association with the prostate cancer metastases. The Adj. *p* is based on the marginally adjusted *p* values by the BH-FDR method.

Gene	Probe ID	Ch	Start	Stop	Adj. *p*	LASSO	MCP
RAB25	2361272	chr1	156041891	156042035	0.003122859	*∗*	
CAMK2N1	2400181	chr1	20810150	20810212	0.003122859	*∗*	*∗*
LOC728875	2432120	chr1	143692898	143692956	0.007211879	*∗*	
AN07	2536262	chr2	242163962	242164581	0.003122859	*∗*	*∗*
FTH1	2590344	chr2	181551038	181551091	0.009379258	*∗*	
RPL7A	3284321	chr10	33483529	33483624	0.007451575	*∗*	*∗*
MKI67	3312502	chr10	129899547	129899701	0.003122859	*∗*	
MALAT1	3377635	chr11	65206468	65206658	0.009379258	*∗*	*∗*
MYBPC1	3428626	chr12	102030464	102030494	0.009379258	*∗*	*∗*
TMP0	3467302	chr12	98943231	98943926	0.008090076	*∗*	*∗*
UBE2C	3887068	chr20	44445472	44445507	0.001041641	*∗*	*∗*
DID01	3913561	chr20	156041891	156042035	0.003122859	*∗*	*∗*

^*∗*^Each gene is identified by the variable selection method.
