# Supplementary material for: High Dimensional Variable Selection with Error Control
Source: Biomed Res Int. 2016 Aug 15;2016:8209453. doi: 10.1155/2016/8209453 (PMC5002494; doi:10.1155/2016/8209453)

Supplementary Files

Table S1| The true positive rate (TP), the false positive rate (FP), the false discovery rate (FDR), the average number of false positive in the final model (ANFP), model size (Size), and the area under the curve (AUROC), the number of filtered truly important variable from FDR and RF (# filter) under the low correlation coefficients, ρ= 0 for the 500 simulations.

screening	method	TP	FP	FDR	ANFP	Size	AUROC	# filter
$FDR_{.05} - ISIS$	LASSO	0.2348 (0.00233)	2e-06 (0)	0.02917 (0.00277)	0.204 (0.01912)	6.074 (0.063)	0.81701 (0.00165)	6.074 (0.063)
	SCAD	0.2348 (0.00233)	2e-06 (0)	0.02917 (0.00277)	0.204 (0.01912)	6.074 (0.063)	0.81701 (0.00165)	
	MCP	0.2348 (0.00233)	2e-06 (0)	0.0283 (0.00274)	0.198 (0.01893)	6.068 (0.06292)	0.81701 (0.00165)	
$FDR_{.20} - ISIS$	LASSO	0.28056 (0.00259)	1.28e-05 (0)	0.13122 (0.00566)	1.284 (0.06609)	8.298 (0.10205)	0.82968 (0.00165)	8.298 (0.10205)
	SCAD	0.28056 (0.00259)	1.28e-05 (0)	0.13092 (0.00565)	1.28 (0.066)	8.294 (0.10184)	0.82965 (0.00165)	
	MCP	0.28056 (0.00259)	1.23e-05 (0)	0.12636 (0.00568)	1.234 (0.06602)	8.248 (0.10092)	0.82978 (0.00165)	
$RF_{1000} - ISIS$	LASSO	0.45512 (0.00257)	0.000116 (0)	0.5053 (0.00279)	11.622 (0.06418)	23 (0)	0.84898 (0.00158)	14.392 (0.06546)
	SCAD	0.45728 (0.00261)	0.000115 (0)	0.50296 (0.00283)	11.568 (0.06514)	23 (0)	0.84888 (0.00157)	
	MCP	0.46192 (0.00269)	0.000114 (0)	0.49791 (0.00293)	11.452 (0.06735)	23 (0)	0.84956 (0.00157)	

( ): standard deviation

Table S2| The true positive rate (TP), the false positive rate (FP), the false discovery rate (FDR), the average number of false positive in the final model (ANFP), model size (Size), and the area under receiver operating characteristic curve (AUROC), the number of filtered truly important variable from FDR and RF (# filter) under the low correlation coefficients, ρ= 0.4 for the 500 simulations.

screening	method	TP	FP	FDR	ANFP	Size	AUROC	# filter
$FDR_{.05} - ISIS$	LASSO	0.19448 (0.00149)	1.8e-06 (0)	0.02953 (0.00327)	0.184 (0.0211)	5.046 (0.04413)	0.76712 (0.00159)	5.052 (0.0448)
	SCAD	0.1936 (0.00149)	1.8e-06 (0)	0.02953 (0.00327)	0.184 (0.0211)	5.024 (0.0443)	0.76702 (0.00159)	
	MCP	0.19088 (0.00154)	1.8e-06 (0)	0.02973 (0.00329)	0.184 (0.0211)	4.956 (0.04561)	0.76658 (0.00159)	
$FDR_{.20} - ISIS$	LASSO	0.22728 (0.00188)	9.9e-06 (0)	0.12385 (0.00588)	0.99 (0.05595)	6.672 (0.07893)	0.77586 (0.00165)	6.690 (0.07975)
	SCAD	0.2248 (0.00179)	9.9e-06 (0)	0.12493 (0.00591)	0.99 (0.05595)	6.61 (0.07741)	0.77574 (0.00165)	
	MCP	0.22152 (0.00185)	9.9e-06 (0)	0.12579 (0.00597)	0.988 (0.05591)	6.526 (0.07863)	0.77527 (0.00165)	
$RS_{1000} - ISIS$	LASSO	0.3428 (0.00246)	0.0001442 (0)	0.62705 (0.00269)	14.412 (0.06232)	22.982 (0.00595)	0.76657 (0.00184)	11.876 (0.07313)
	SCAD	0.33832 (0.0024)	0.0001452 (0)	0.63175 (0.00263)	14.514 (0.06108)	22.972 (0.00739)	0.76576 (0.00185)	
	MCP	0.33496 (0.00236)	0.000146 (0)	0.63542 (0.00258)	14.598 (0.06007)	22.972 (0.00739)	0.76553 (0.00184)	

(): standard deviation

Table S3| Average true regression coefficients for the 25 variables

Variable name	True regression coefficient
g01	1.12939
g02	0.28505
g03	-1.45882
g04	0.12632
g05	-0.35443
g06	-1.01840
g07	1.65116
g08	-0.76401
g09	0.84540
g010	-0.69254
g11	-0.07449
g12	0.96375
g13	0.23261
g14	-0.42302
g15	0.47535
g16	-0.12398
g17	-0.33047
g18	-0.87834
g19	1.57071
g20	-1.23074
g21	0.73234
g022	-0.55067
g23	1.17932
g24	0.96817
g25	-0.72762

Table S4| AUROC scores, the corresponding confidence interval, and standard error for LASSO, SCAD, and MCP methods on six different screening scenarios with prostate cancer data.

Method		AUROC	Confidence Interval		S.E
Screening	Variable Selection		lower	upper	
$FDR_{0.01} - ISIS$	LASSO	0.74622	0.67467	0.81777	0.03650
	SCAD	0.74585	0.67429	0.8174	0.03651
	MCP	0.76437	0.69508	0.83366	0.03535
$FDR_{0.05} - ISIS$	LASSO	0.69712	0.61929	0.77495	0.03971
	SCAD	0.71405	0.63739	0.79072	0.03911
	MCP	0.68335	0.60335	0.76335	0.04081
$FDR_{0.20} - ISIS$	LASSO	0.70906	0.63178	0.78634	0.03943
	SCAD	0.68944	0.61178	0.76711	0.03962
	MCP	0.71539	0.63801	0.79278	0.03948
RF_{1000}	LASSO	0.73586	0.66149	0.81024	0.03794
	SCAD	0.73757	0.66269	0.81245	0.03820
	MCP	0.73757	0.66269	0.81245	0.03820
RF_{2000}	LASSO	0.73684	0.66227	0.81140	0.03804
	SCAD	0.70626	0.62894	0.78357	0.03944
	MCP	0.71929	0.64044	0.79815	0.04023
RF_{4000}	LASSO	0.69273	0.61330	0.77216	0.04052
	SCAD	0.72100	0.64440	0.79759	0.03907
	MCP	0.69480	0.61450	0.77511	0.04097

S.E was estimated by bootstrapping approach.

Figure S1| Selection frequencies of each of the 25 variables across the LASSO, the SCAD, and the MCP during 500 simulations with $\rho = 0$. The x-axis depicts the names of the variables, and the y-axis is the frequency of variables selected out of 500 simulations. The variables not depicted on the x-axis in Figure 2 did not have any counts. Each of the three methods is identified by color in legend.

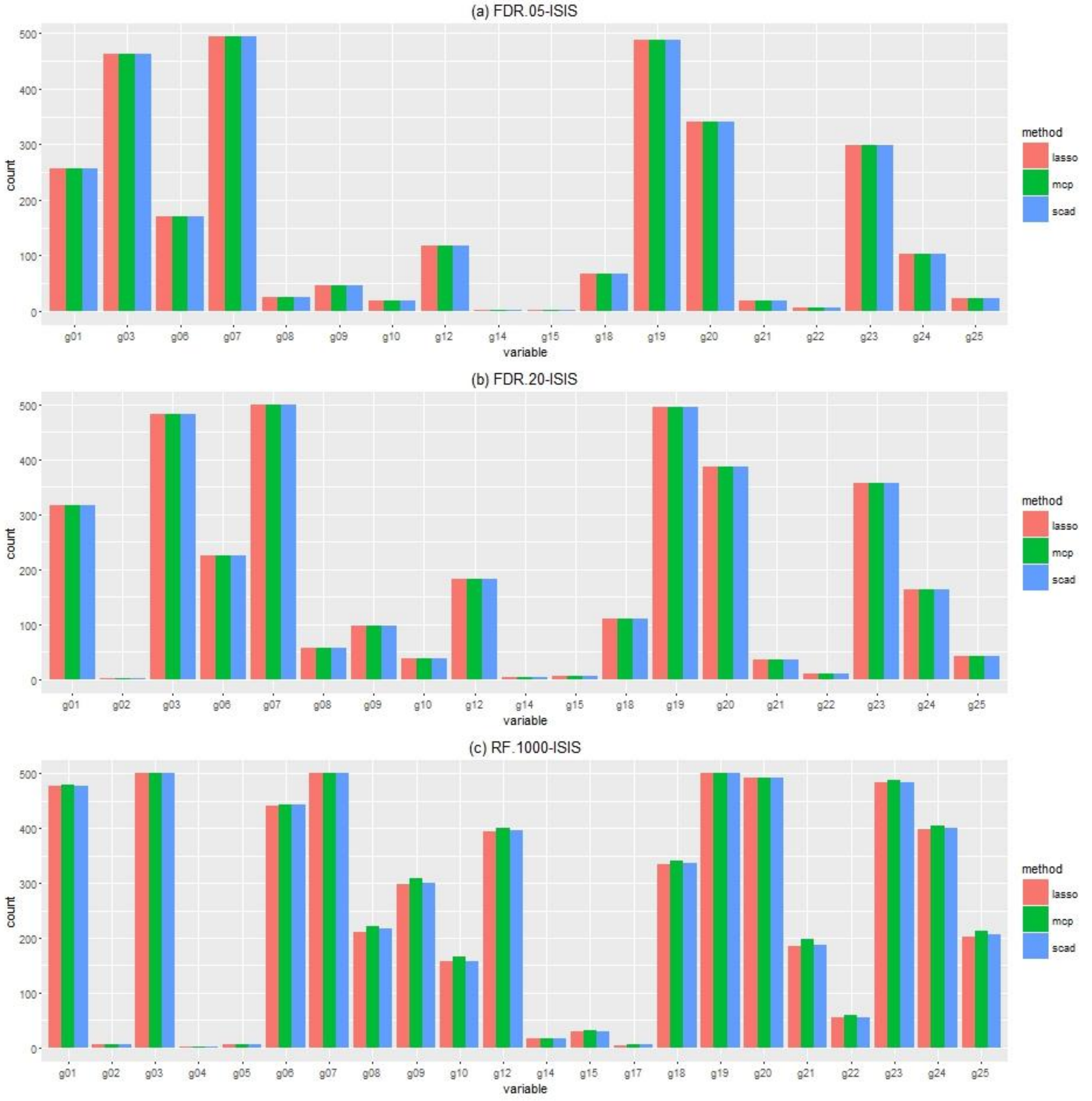

Figure S2| Selection frequencies of each of the 25 variables across the LASSO, the SCAD, and the MCP during 500 simulations with $\rho = 0.4$. The x-axis depicts the names of the variables, and the y-axis is the frequency of variables selected out of 500 simulations. The variables not depicted on the x-axis in Figure 2 did not have any counts. Each of the three methods is identified by color in legend.

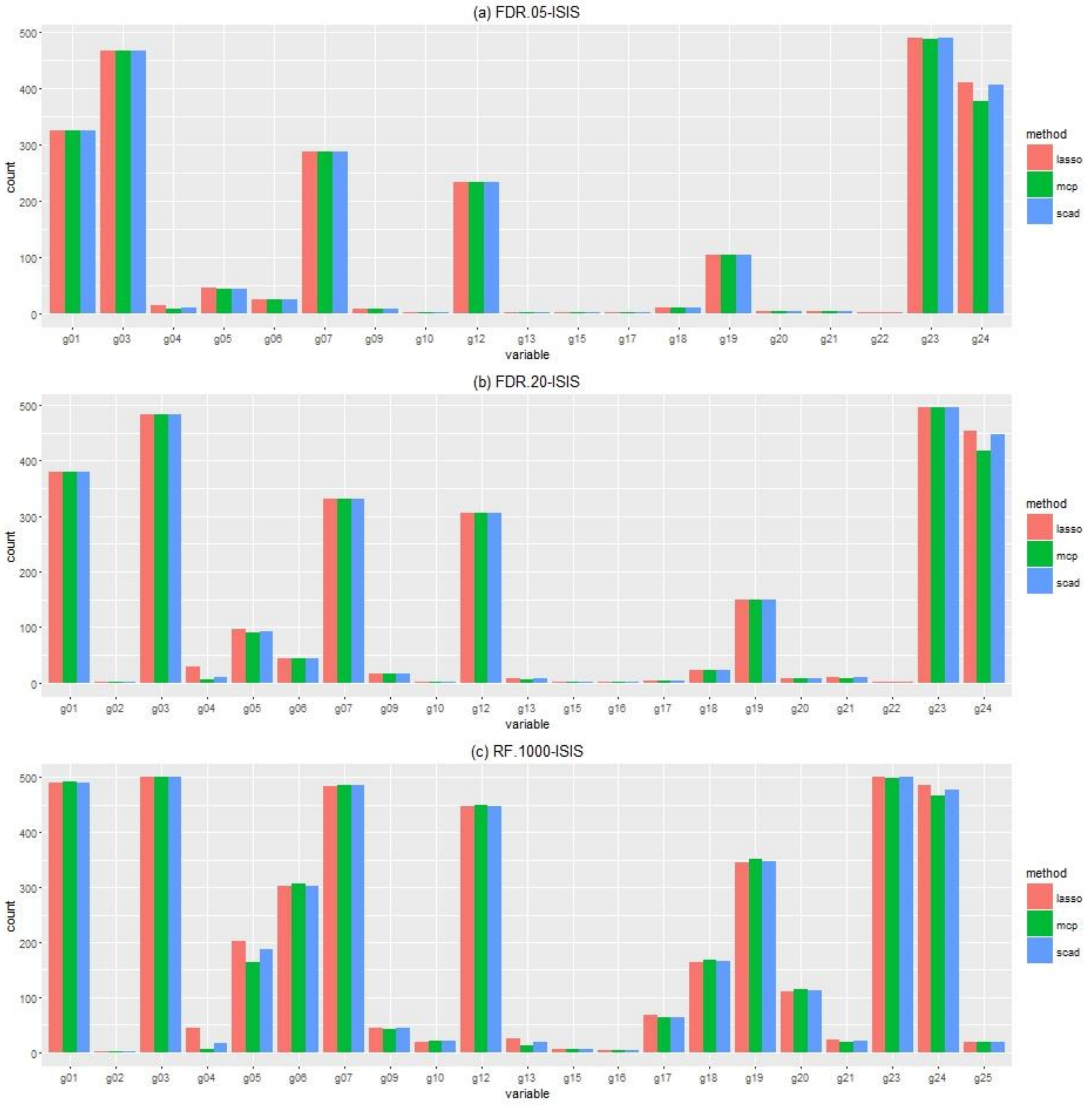

Figure S3| First column is the box plots of the AUROC scores under $\rho = 0$. x-axis is the name of variable selection methods ,and y–axis is the AUROC scores. Second column is the box plots of the corresponding false discovery rate, and y-axis the false discovery rate during 500 simulations.

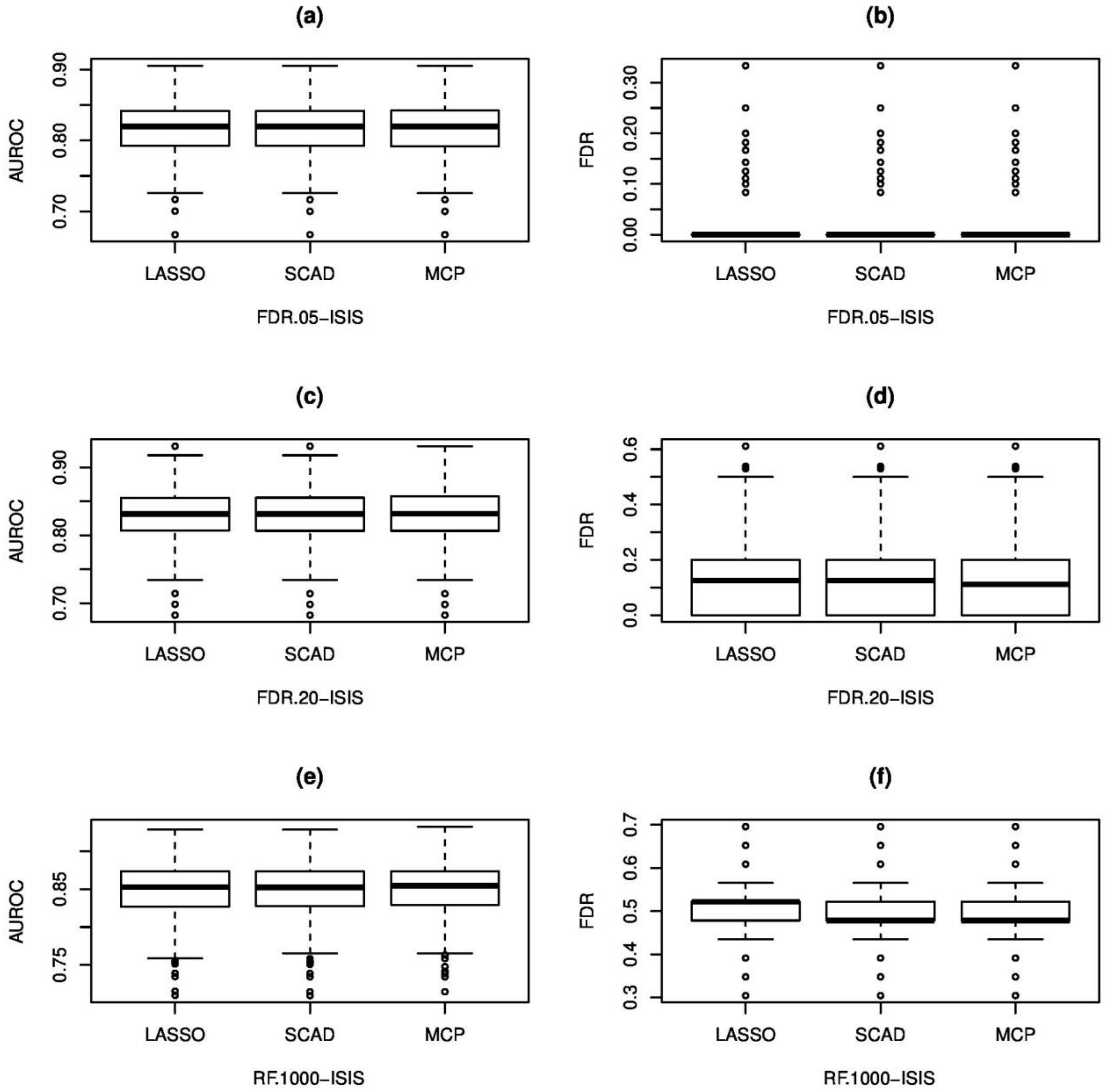

Figure S4| First column is the box plots of the AUROC scores under $\rho = 0.4$. x-axis is the name of variable selection methods ,and y –axis is the AUROC scores. Second column is the box plots of the corresponding false discovery rate, and y-axis the false discovery rate during 500 simulations.

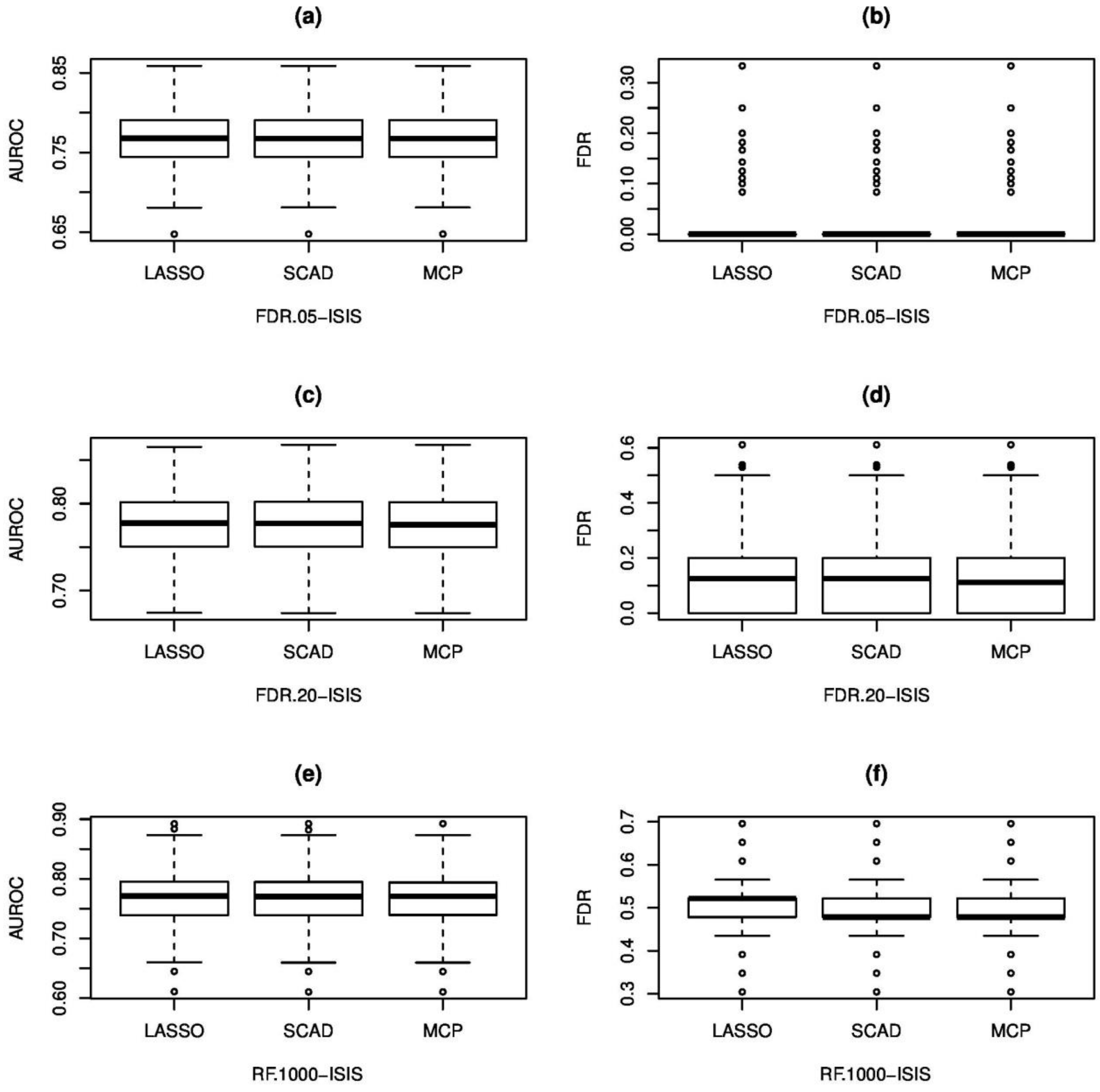

Figure S5| Boxplots of the expressions of 12 detected genes on the prostate cancer. Both AN07 and MYBPC1 are the top down-regulated whereas the rest of them are the top up-regulated. The red color is no metastasis and another is metastasis.

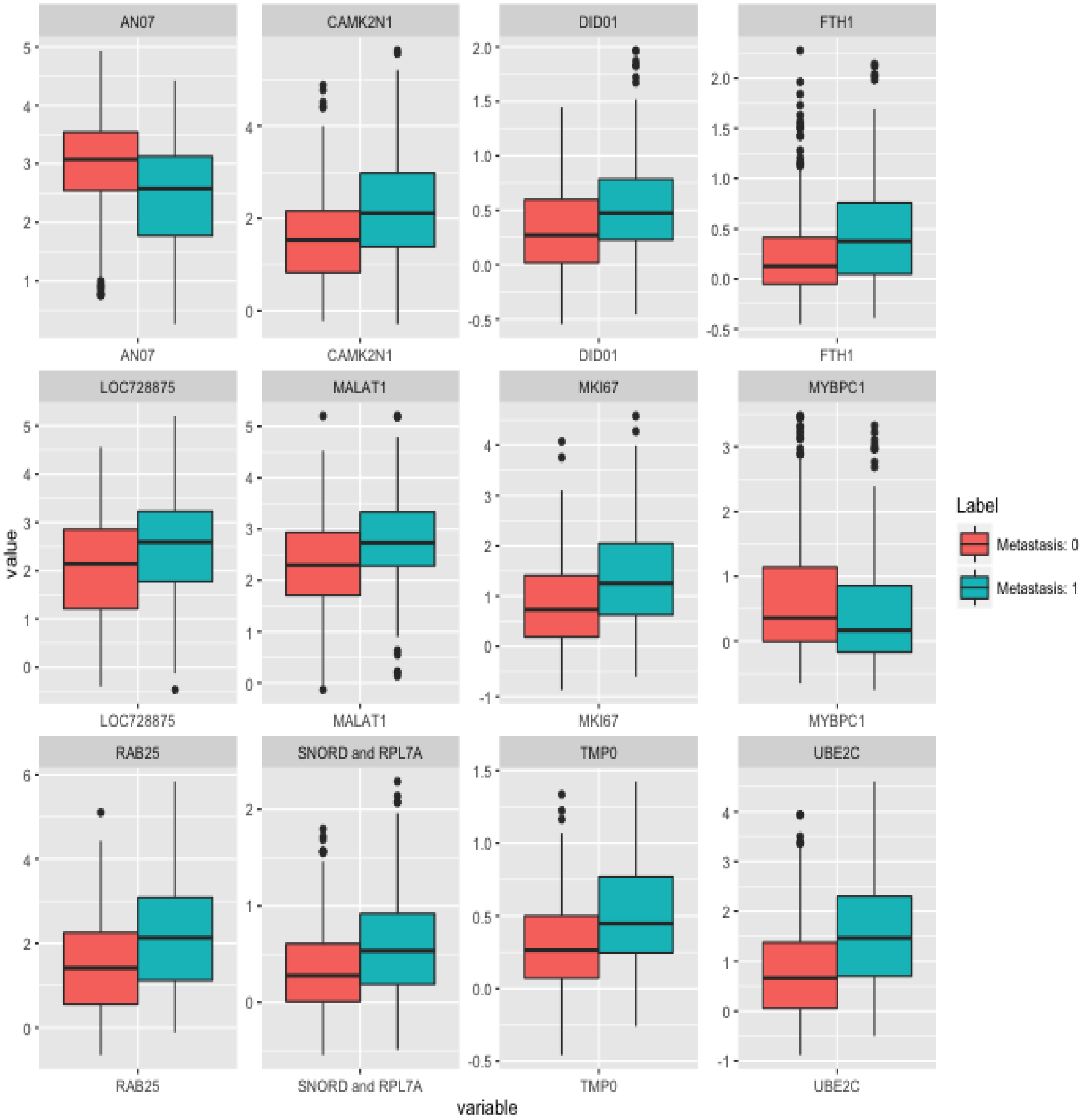

Supplement: Supplementary file 1 — Supplementary file is composed of the six parts of additional information that were not explained in the manuscript. Table S1 and S2 are the comparison of the performance for random filtering screening and FDR screenings with independent and moderate correlation ρ = {0, 0.4}, respectively in the simulation studies. Table S3 represents average true regression coefficients for the 25 variables through 500 replicates. Table S4 is comparison of area under the curve (AUC) and the corresponding 95% confidence interval with three popular variable selection methods (LASSO, SCAD, and MCP) for random screenings (1000,2000, and 4000) and FDR screenings at α = 0.01,0.05, and 0.20. Figure S1 and S2 shows the selection frequencies of each of the 25 variables across the LASSO, SCAD, and the MCP during 500 simulations with independent and moderate correlation ρ = {0, 0.4}. Figure S3 and S4 present AUC scores and the corresponding mean false discovery rate during the simulations with ρ = {0, 0.4}. Finally, Figure S5 shows boxplots of the expressions of 12 identified genes on metastasis in the prostate cancer. [file 8209453.f1.pdf]
